# Complete genome sequences of new serotypes of bluetongue virus (BTV) and epizootic hemorrhagic disease virus (EHDV) isolated from a single alpaca in South Africa

**DOI:** 10.1128/mra.00265-26

**Published:** 2026-06-15

**Authors:** Isabella M. Wright, René G. P. van Gennip, Christiaan A. Potgieter, Piet A. van Rijn

**Affiliations:** 1 Research and Development Section, Deltamune (Pty) Ltd, Pretoria, South Africa; 2 Virology Division, ARC, Onderstepoort Veterinary Institute 71909, Onderstepoort, South Africa; 3 Department of Virology and Molecular Biology, Wageningen Bioveterinary Research 4507 , Lelystad, the Netherlands; Katholieke Universiteit Leuven, Leuven, Belgium

**Keywords:** orbiviruses, serotype, bluetongue virus, epizootic hemorrhagic disease virus

## ANNOUNCEMENT

We announce complete genome sequences of bluetongue virus and epizootic hemorrhagic disease virus isolated from a single alpaca in South Africa in 2008. Similar to recently discovered new serotypes, phylogenetic analyses suggest that both isolates are new serotypes of these orbivirus species (genus *Orbivirus*, family *Sedoreoviridae*, order *Reovirales*).

**Fig 1:**
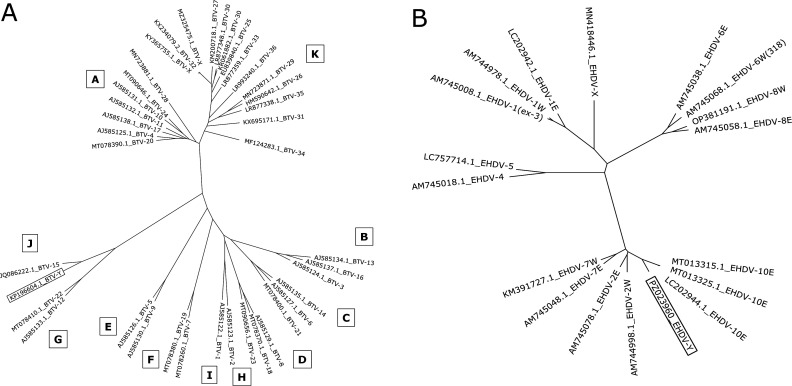
Genotyping of Seg-2 of BTV-Y(ALP8) and EHDV-Y(ALP9). Nucleotide sequences of Seg-2 of representative BTV and EHDV serotypes were aligned using ClustalW (DNASTAR, version 17). Phylogenetic relationships were computed by maximum likelihood (RAxML) using 100 bootstrap replicates, and the unrooted tree was viewed by TreeViewer. Accession numbers of Seg-2 nucleotide sequences of recognized and putative new serotypes are indicated. Untyped viruses are indicated by X and Y (this announcement, boxed). (**A**) Seg-2 nucleotypes are indicated by A to K. BTV topotypes are not included for readability. (**B**) Topotypes of each of the EHDV serotypes are indicated by the extension of the serotype number with E (eastern) or W (western).

Orbiviruses are nonenveloped, multiprotein-layered viruses containing a 10-segmented genome of double-stranded RNA (1). Bluetongue virus (BTV) and epizootic hemorrhagic disease virus (EHDV) are transmitted by *Culicoides* biting midges, causing disease in ruminants and camelids (2, 3). BTV and EHDV consist of many serotypes. BTV serotypes 1–24 and EHDV cause notifiable diseases (4). Serotypes are determined by virus neutralization tests and serum neutralizing tests (serotyping) (5). VP2 protein encoded by genome segment 2, Seg-2[VP2], is the main target for serotype-specific neutralizing antibodies (6). Serotypes are grouped by some cross-neutralization (7). These groups match with the phylogenetic analysis of Seg-2[VP2] (genotyping) and are named nucleotypes “A” to “K” (8, 9). Since 2008, novel serotypes (putatively named BTV-24+) have been discovered (10–17), which is mainly based on inter-serotype genetic variation (8). Except for serotype 28, all recently discovered BTV serotypes are members of nucleotype “K” (Fig. 1A). BTV-X appeared as a variant of BTV-32. Intra-serotype variation can be associated with their geographic origin (topotypes) (18). Initially, EHDV encompassed nine serotypes; however, EHDV-3 (ex-3) and EHDV-9 (318) have been regrouped into serotypes 1 and 6, respectively (19) (Fig. 1B). Detailed genotyping of Seg-2 clearly distinguished eastern and western (W) topotypes within each of the EHDV serotypes (Fig. 1B). Recently, EHDV-10E and the putative new EHDV-X were discovered in China and Japan, respectively (20, 21). Serotyping of putative new serotypes is mostly impossible since virus isolates or suitable antibody-positive sera are not available.

Two orbivirus isolates, ALP8 and ALP9, were isolated from a single alpaca (*Vicugna pacos*) in 2008 (22). The following methods have been extensively described in detail (23, 24). Briefly, dsRNA was purified using TRI Reagent–LS (Molecular Research Center), LiCl precipitation of ssRNA, and extraction by MinElute Gel extraction columns used for dsDNA purification (Qiagen). A 5′-phosphorylated primer forming a loop (TibMolbiol) was ligated to dsRNA, similarly purified again (Qiagen), reverse transcribed with AMV reverse transcriptase (Invitrogen), and PCR-amplified using a second primer and Phusion polymerase (Finnzymes). cDNA was purified (PCR Purification Kit, Qiagen) and sequenced on a GS20 sequencer (Roche 454). Files were loaded into Seqman (DNASTAR, Lasergene v8.1.2) for *de novo* sequence assembly using default parameters. A 40-fold coverage allowed sequencing of complete genomes. Contig sequences were manually checked to generate consensus sequences. ALP8 (BT 57/08) segments revealed the highest similarities with BTV-15, except for Seg-2 (Table 1A). The highest inter-serotype similarity of 72.6% for Seg-2 and the very weak neutralization of ALP8 (BT57/08) by only serotype 15 serum (as determined previously) indicated a new serotype, 37 (8, 22). Clearly, genotyping of Seg-2 (KP196604) grouped ALP8 (BTV-Y) to nucleotype “J” (Fig. 1A). Segments of ALP9 showed the highest similarities with EHDV-6 or 7, except for Seg-2 and Seg-6 (Table 1B). The highest similarity (74.3%) of Seg-2 was with EHDV-10E/ON-4/B98 (LC202944). Lack of cross-neutralization by sera against EHDV serotypes 1–8 suggests another serotype (EHDV-Y) (22), while the low intra- and inter-serotype similarity of Seg-2 also suggests another serotype or a W topotype of serotype 10 (EHDV-10W) (19) (Fig. 1B).

**TABLE 1:** Similarity per genome segment of BTV-Y(ALP8) and EHDV-Y(ALP9)^*a*^

A				
Genome segment	BTV-Y/ALP8 accession no.	Highest similarity	Accession no.	BTV isolate
Seg-1[VP1]	KP196603	98.8	KP820978	BTV-15/ISR2006/11
Seg-2[VP2]	KP196604	72.6	JQ086222	BTV-15/DPP192
Seg-3[VP3]	KP196605	98.5	KP821220	BTV-15/ISR2006/11
Seg-4[VP4]	KP196606	98.8	KP821340	BTV-15/ISR2006/11
Seg-5[NS1]	KP196607	98.8	KP821460	BTV-15/ISR2006/11
Seg-6[VP5}	KP196608	90.7	JX272474	BTV-15/23/8/65
Seg-7[VP7]	KP196609	97.2	DQ465027	BTV-15/RSArrrr
Seg-8[NS2]	KP196610	98.6	KP821822	BTV-15/ISR2006/11
Seg-9[VP6/NS4]	KP196611	98.3	KP821942	BTV-15/ISR2006/11
Seg-10[NS3/NS3a/NS5]	KP196612	98.0	ON087705	BTV/ISR-2237/18

B				
Genome segment	EHDV-Y/ALP9 accession no.	Highest similarity	Accession no.	EHDV isolate
Seg-1[VP1]	PZ023959	98.8	HM636907	EHDV-6/M44/96
Seg-2[VP2]	PZ023960	74.3	LC202944	EHDV-10/ON-4/B/98
Seg-3[VP3]	PZ023961	99.2	HM636909	EHDV-6/M44/96
Seg-4[VP4]	PZ023962	98.6	HM636910	EHDV-6/M44/96
Seg-5[NS1]	PZ023963	99.2	HM636911	EHDV-6/M44/96
Seg-6[VP5}	PZ023964	82.7	LC202962	EHDV-2 KS-7E/13
Seg-7[VP7]	PZ023965	95.0	KM391739	EHDV-7/ISR2006/02
Seg-8[NS2]	PZ023966	98.7	HM636914	EHDV-6/M44/96
Seg-9[VP6/NS4]	PZ023967	98.3	KM391747	EHDV-7/ISR2006/06
Seg-10[NS3/NS3a/NS5]	PZ023968	99.4	HM636916	EHDV-6/M44/96

^*a*^ Consensus nucleotide sequences of complete genome segments 1 to 10 of BTV-Y(ALP8) (A) and EHDV-Y(ALP9) (B) are deposited to GenBank. Each genome segment was compared to available sequences in the GenBank database by the BLASTN tool of the NCBI website. Accession numbers of nucleotide sequences of indicated virus isolates with the highest similarity are shown.

## Data Availability

Raw data of ALP8 (BTV-Y) and ALP9 (EHDV-Y) are available in BioProjects PRJNA1463036 and PRJNA1463056, respectively. Nucleotide sequences of genome segments of ALP8 (BTV-Y) and ALP9 (EHDV-Y) are deposited in GenBank under the accession numbers listed in Table 1.
